# The Role of the Gut Microbiota in Vascular Physiology and Health

**DOI:** 10.3390/ijms262311553

**Published:** 2025-11-28

**Authors:** Maria Adriana Neag, Liviu-Ștefan Moacă, Andrada-Larisa Deac, Dan Claudiu Măgureanu, Damiana-Maria Vulturar, Doina Adina Todea, Diana Gherman, Anca Dana Buzoianu, Claudia Diana Gherman, Florentina Claudia Militaru

**Affiliations:** 1 Department of Pharmacology, Toxicology and Clinical Pharmacology, “Iuliu Hațieganu” University of Medicine and Pharmacy, 400012 Cluj-Napoca, Romania; 2 Department of Surgery-Practical Abilities, “Iuliu Hațieganu” University of Medicine and Pharmacy, 400337 Cluj-Napoca, Romania; 3 Department of Pneumology, “Iuliu Hațieganu” University of Medicine and Pharmacy, 400332 Cluj-Napoca, Romania; 4 Department of Radiology, “Iuliu Hațieganu” University of Medicine and Pharmacy, 400347 Cluj-Napoca, Romania

**Keywords:** nutraceuticals, microbiota, cardiovascular disease, SCFA, inflammation, vascular health

## Abstract

Cardiovascular diseases (CVD) are the leading cause of global morbidity and mortality. There are many well-known risk factors (dyslipidemia, atherosclerosis, diabetes mellitus, obesity) for these diseases, but there are also other factors with important implications in the pathogenesis of vascular diseases (VD). One of these is the gut microbiome, a metabolically active “organ” with many roles in physiological processes that are linked to VD. Moreover, in recent years, the microbiota has been studied as a “target” in the treatment of various diseases, from gastrointestinal diseases to cardiovascular or even neuropsychiatric diseases. Thus, nutraceuticals may have an important role in modulating the gut microbiota and preventing or treating VD. The aim of this review is to highlight the relationship between the intestinal microbiota and the development and progression of cardiovascular diseases, as well as the possible intervention by using nutraceuticals (vitamins, minerals, dietary supplements) to positively modulate the intestinal microbiota and thus to reduce the risk of VD.

## 1. Introduction

Cardiovascular diseases (CVD) represent the leading cause of death worldwide with more than 18 million deaths annually, accounting for over 30% of global deaths [1]. According to the data from Global Burden Disease (GBD) study published in 2020, the prevalence of CVD almost doubled between 1990–2019, from 271 million to 523 million cases [1]. Ischemic heart disease represents the leading cause of death worldwide with 9 million deaths every year [2]. Risk factors for CVD can be categorized as non-modifiable and modifiable. The factors that cannot be changed consist of age, male sex, genetics and family history of CVD, and ethnicity [3]. Modifiable risk factors include hypertension, dyslipidemia, obesity or overweight, type 2 diabetes mellitus, smoking and alcohol consumption, unhealthy diet, physical activity, and chronic stress [3]. CVD includes hypertension, atherosclerosis, ischemic heart failure, stroke, aortic valve disease, and heart failure.

The human microbiome refers to the collective genetic material of microorganisms (microbiota) residing in specific areas of the body [4]. These microorganisms colonize various anatomical sites, including the skin, mucosa, gastrointestinal tract, respiratory tract, urogenital tract, and mammary glands, but with the highest amount harbored in the gut [4,5]. They create a complex and distinct ecosystem that adapts to the unique environmental conditions of each niche. The microbiota of every person consists of 10–100 trillion symbiotic microbial cells and the genes that these cells harbor [6]. The gut microbiota is composed of bacteria, fungi, viruses, archaea, and phages [7]. The primary bacterial species contained by the gut include *Bacteroidetes*, *Actinobacteria*, *Firmicutes*, *Proteobacteria*, *Verrucomicrobia*, and *Fusobacteria* [8]. *Bacteroidetes* and *Firmicutes* encompass 90% of gut microbiota [9]. The function of gut microbiota includes fermentation of indigestible fibers, vitamin synthesis, energy production via short-chain fatty acids (SCFAs), maintenance and regulation of the epithelial intestinal mucosa barrier [10].

The term “nutraceutical” is derived from two words, “nutrition” and “pharmaceutical”, and was defined by Stephen DeFelice thirty-five years ago as “a food or part of a food that provides medical or health benefits” in order to prevent and/or treat a disease [11,12]. There are similarities between the definition of nutraceuticals and food supplements; however, nutraceuticals include single or combinations of prebiotic and probiotic foodstuffs and food that are used for medical purposes, and the latter are used single or in a mixture of vitamins, protein, mineral, and herbal products to compensate the deficiency of micronutrients and macronutrients [12,13]. Based on the previous characteristics, nutraceuticals are also referred to as “pharma-food” and are used “beyond the diet, and before drugs” with the role of preventing or delaying the onset of certain asymptomatic diseases, such as hypercholesterolemia or hypertriglyceridemia [12]. Nutraceuticals from food sources are natural and can be classified as dietary fiber, prebiotics, probiotics, carotenoids, polyunsaturated fatty acids, polyphenols, antioxidants vitamins, and spices [14]. Nutraceuticals encompass a wide range of therapeutic areas, including arthritis relief, cold and cough management, sleep disorders, digestive health, and the prevention of certain cancers [15]. They also play a role in managing osteoporosis, regulating blood pressure and cholesterol levels, providing pain relief, and supporting mental health conditions such as depression, as well as diabetes management [15].

Consumption of an unhealthy diet is an important risk factor for the pathogenesis of CVD as it increases triglyceride and cholesterol and induces systemic inflammation [16]. Food and nutrients are essential for gut-microbiota, so feeding healthy and proper nutrients is beneficial for the body. Food is primarily digested and absorbed in the intestine, and nutrients are transported to the liver via the portal vein. Metabolites produced by gut microbiota such as trimethylamine N-oxide (TMAO), phenylacetic acid, lipopolysaccharide (LPS), peptidoglycan, and SCFAs are absorbed through intestinal epithelial cells and carried to the liver [17]. TMAO, LPS, and phenylacetic acid are risk factors for CVD, while others such as SCFAs possess an anti-inflammatory role [17]. High levels of TMAO are responsible for arterial damage and create a state of chronic micro-inflammation therefore increasing the cardiovascular risk by 60% [18]. SCFAs are produced by fermentable fibers. These fatty acids stimulate the production of mucin improving the barrier effect of the intestinal mucosa, and decreasing the passage of cells and molecules in the circulation, all of these leading to a reduction in the body’s inflammation [18]. Another anti-inflammatory mechanism of SCFA consists of the inhibition of nuclear factor NF-κB and the activity of histone deacetylase [19]. Both these mechanisms also suppress the release of TNF-α [19]. All these anti-inflammatory effects suggest a possible preventive action in atherosclerosis and improve the clinical manifestation of CVD. Microbiota can also interfere with the bioavailability of antioxidants which can increase their protective effect [18]. Several nutraceuticals are known to interact with the microbiota. Prebiotics from fibers promote the growth of beneficial gut microbiota, which can have a downstream effect on metabolism and inflammation [20]. A diet rich in fiber can modify gut microbiota to increase acetate-producing bacteria, which has a cardioprotective effect [20]. Live bacteria or probiotics can improve gut health through a protective effect, reduce inflammation, and enhance immune function [21]. Most probiotics contain *Bifidobacteria*, *Lactobacilli*, *Lactococci*, and *Streptococci*. Some polyphenols found in fruits and vegetables such as flavonoids, resveratrol, and ellagic acid have been shown to modulate the gut microbiota, promote the growth of beneficial bacteria, and reduce CVD risk [10].

## 2. The Role of Microbiota

The human gut microbiota plays a fundamental role in maintaining host homeostasis by orchestrating a multitude of physiological functions across various organ systems [22,23]. As a complex and dynamic microbial ecosystem, it has co-evolved with the host, significantly influencing immunological [24], metabolic [25,26], structural [27], and neurological [28,29] processes. The development of the gut microbiota begins at birth, with microbial colonization shaped by factors such as mode of delivery, early feeding practices, genetic predisposition, and environmental exposures. Throughout life, its composition remains relatively stable yet adaptable to dietary changes, medical interventions, and disease states [30].

Functionally, the gut microbiota serves as a key modulator of immune system activity. It plays an essential role in the maturation and function of gut-associated lymphoid tissue (GALT), shaping innate and adaptive immune responses [31]. Microbial antigens stimulate the differentiation of regulatory T cells [32], influence antigen-presenting cell (APC) activity [33], and modulate the production of inflammatory and anti-inflammatory cytokines [34]. Dysbiosis, or microbial imbalance, has been implicated in autoimmune diseases, chronic inflammatory conditions, and hypersensitivity reactions, underscoring the microbiota’s role in immune regulation [35]. Metabolically, the gut microbiota contributes to host energy balance through the fermentation of non-digestible carbohydrates, producing SCFAs such as acetate, propionate, and butyrate [36]. These SCFAs serve as critical energy substrates for colonocytes, modulate glucose and lipid metabolism, and exert anti-inflammatory properties within the gut and systemically [37]. Additionally, gut microbes facilitate the synthesis of essential vitamins (such as vitamin K, and B vitamins), contribute to amino acid metabolism, and participate in bile acid biotransformation, all of which are essential for maintaining metabolic homeostasis [38]. Alterations in microbial composition have been associated with metabolic disorders such as obesity [39], insulin resistance [40], and non-alcoholic fatty liver disease [41]. Structurally, the gut microbiota supports the integrity of the intestinal epithelial barrier, regulating mucin production, tight junction assembly, and antimicrobial peptide secretion [42]. A well-maintained epithelial barrier prevents the translocation of pathogenic microbes and endotoxins into the systemic circulation, reducing the risk of chronic inflammation and associated diseases such as inflammatory bowel disease (IBD), cardiovascular disease, and neurodegenerative disorders [43]. Disruptions to this barrier, often termed “leaky gut syndrome,” have been linked to systemic inflammatory conditions and metabolic dysfunctions [35]. Neurologically, the gut microbiota exerts influence through the microbiota–gut–brain axis, a bidirectional communication network that integrates neural, endocrine, and immune pathways [44]. Microbial metabolites, including SCFAs, neurotransmitters (e.g., serotonin, gamma-aminobutyric acid), and microbial-derived peptides, modulate neurodevelopment, cognitive function, and emotional well-being. Dysbiosis has been associated with neurological and psychiatric conditions such as depression, anxiety, autism spectrum disorders, and neurodegenerative diseases, highlighting the importance of gut microbial balance in mental health [45]. The recognition of gut microbiota’s functional importance has led to the development of microbiota-targeted therapeutic strategies. These include prebiotics, probiotics, synbiotics, postbiotics, and fecal microbiota transplantation (FMT), all of which aim to restore eubiosis and improve host health outcomes [46]. Advances in precision medicine and microbiome-based diagnostics further enhance our ability to personalize interventions for gastrointestinal, metabolic, immunological, and neuropsychiatric disorders [47].

## 3. Microbiota Dysbiosis and Its Implications for Vascular Health

Researchers have sought to identify the main mechanism through which dysbiosis can lead to this wide spectrum of complications in recent years. One hypothesis is that dysbiosis impacts vascular structural and functional impairment—changes that could underlie all pathologies associated with dysbiosis.

### 3.1. Dysbiosis and Atherosclerosis

**Figure 1:**
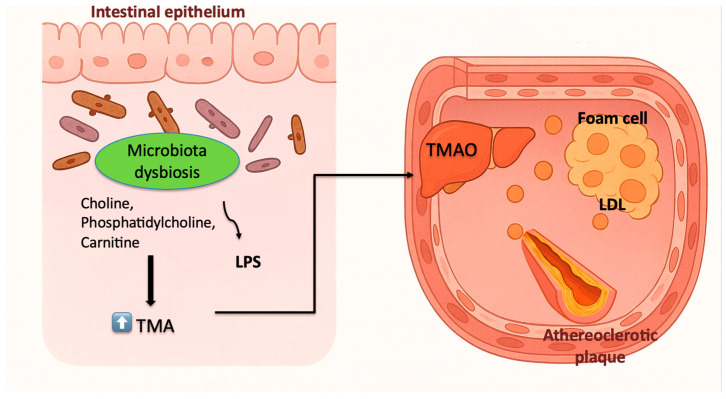
Microbiota dysbiosis and its metabolic contribution to atherosclerosis.

In this regard, many studies have investigated the potential link between dysbiosis and atherosclerosis. Atherosclerosis is a chronic progressive disease characterized by the accumulation of lipids, inflammatory cells, and fibrous elements in the arterial wall, leading to atherosclerotic plaques [48]. This process results in arterial narrowing and reduced blood flow, increasing the risk of major cardiovascular events such as myocardial infarction and stroke [49]. In this regard, it has been observed that intestinal dysbiosis is associated with increased levels of trimethylamine (TMA), a compound derived from microbial metabolism of dietary choline, phosphatidylcholine, and carnitine, that, once absorbed into the portal circulation, is oxidized in the liver to TMAO, a molecule with strong pro-inflammatory and pro-atherogenic effects [50,51]. To determine the primary cause of increased TMA production, multiple microbiota studies have been conducted, identifying *Streptococcus sanguinis*, *Acinetobacter*, *Escherichia coli*, *Klebsiella pneumoniae*, *Citrobacter*, *Shigella*, *Achromobacter*, *Sporosarcina*, and *Desulfovibrio* spp. as the main causative bacteria [52]. Besides these bacterial populations, another major precursor of dysbiosis that can lead to elevated TMAO levels is a diet rich in animal fat, coupled with low or absent consumption of plant-based products [53]. Another metabolite frequently identified in association with intestinal dysbiosis is LPS, which exhibits elevated serum levels [54]. These metabolites play a significant role in the atherosclerotic process both directly, by stimulating the release of low-density lipoprotein (LDL) cholesterol from foam cells, and indirectly, through their pro-inflammatory and pro-oxidative effects throughout the body, which further promote the oxidation of LDL cholesterol within the vascular bed [55] (see Figure 1) Bile acids are another important gut metabolite that was observed to be implicated in the progression of atherosclerosis. Therefore, the disruptions in insulin signaling can lead to increased production of 12α-hydroxylated bile acids, which are associated with a higher risk of developing atherosclerotic disease [56]. Additionally, Baf60a, a transcriptional coactivator, has been identified as a key factor linking high-fat and high-cholesterol diets to elevated plasma cholesterol and plaque formation. Studies have shown that inhibiting Baf60a specifically in hepatocytes reduces bile acid synthesis, lowers intestinal cholesterol absorption, and ultimately mitigates diet-induced atherosclerosis in experimental models [57].

The Figure 1 illustrates how intestinal dysbiosis promotes atherosclerosis through microbial metabolites. Dietary choline, phosphatidylcholine, and carnitine are converted by gut bacteria into trimethylamine (TMA), which is then oxidized in the liver to TMAO—a compound with pro-inflammatory and pro-atherogenic effects. Dysbiosis also increases circulating LPS levels, contributing to endothelial dysfunction and LDL accumulation. Together, these processes promote foam cell formation and atherosclerotic plaque development within the arterial wall.

### 3.2. Dysbiosis and Endothelial Dysfunction

**Figure 2:**
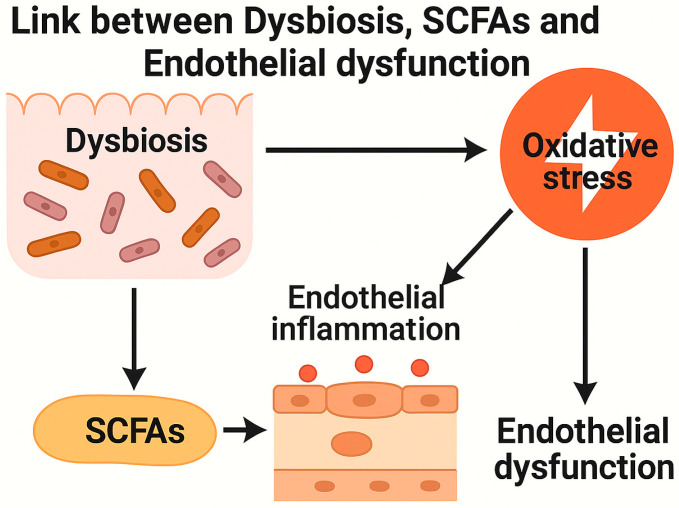
Schematic representation of the link between dysbiosis, short-chain fatty acids (SCFAs), and endothelial dysfunction.

The imbalance of gut microbiota also plays a critical role in endothelial dysfunction by promoting systemic inflammation, oxidative stress, and neurohumoral dysregulation, factors that collectively contribute to vascular impairment and hypertension [58] (a schematic representation is in the Figure 2). One of the key mechanisms involves the depletion of SCFA-producing bacteria, leading to reduced levels of acetate, propionate, and butyrate. These metabolites exert protective vascular effects by activating G-protein-coupled receptors (GPR41/43), enhancing nitric oxide (NO) bioavailability, and mitigating oxidative stress [59]. Furthermore, SCFAs inhibit histone deacetylases (HDACs), particularly HDAC3, thereby downregulating vascular cell adhesion molecule-1 (VCAM-1) expression, which reduces monocyte adhesion to the endothelium and suppresses foam cell formation—a crucial step in atherosclerosis progression [60]. Additionally, butyrate has been shown to counteract endothelial dysfunction induced by angiotensin II by preserving NO bioavailability and preventing excessive vasoconstriction [59]. Another major contributor to endothelial dysfunction in dysbiosis is the increased gut permeability, which facilitates the translocation of bacterial endotoxins such as LPS. LPS-mediated activation of toll-like receptor 4 (TLR4) triggers the release of pro-inflammatory cytokines, including interleukin 6 (IL-6) and interleukin 8 (IL-8), further exacerbating vascular inflammation and arterial stiffness [58]. Moreover, butyrate has demonstrated anti-atherogenic properties by inhibiting the activation of the NLRP3 inflammasome, thereby reducing oxidative stress and limiting neointima formation in endothelial cells [61]. Given the strong link between dysbiosis and vascular dysfunction, therapeutic strategies aimed at restoring microbial balance, such as SCFA supplementation, probiotics, and high-fiber diets, offer promising avenues for improving endothelial health and reducing cardiovascular risk.

### 3.3. Dysbiosis and Arterial Hypertension

The disruption of gut microbiota homeostasis contributes to endothelial dysfunction, systemic inflammation, and neurohumoral dysregulation, all of which are critical in the development of hypertension [62]. One of the primary mechanisms through which dysbiosis affects vascular health and leads to hypertension is the alteration of SCFA production [63]. Since SCFAs play a crucial role in endothelial function, as discussed earlier, their depletion in dysbiosis also contributes to increased vascular stiffness and hypertension. Dysbiosis leads to a reduction in SCFA-producing bacteria, thereby impairing endothelial-dependent vasodilation and increasing vascular stiffness, which predisposes individuals to hypertension [64]. Additionally, an altered gut microbiome promotes systemic inflammation through increased gut permeability and translocation of LPS, which activate TLR4-mediated pro-inflammatory pathways, leading to cytokine release (TNF-α, IL-6) and exacerbation of vascular dysfunction [65]. Dysbiosis further influences blood pressure regulation by modulating the renin-angiotensin system (RAS), as microbial imbalances are associated with elevated renin expression and angiotensin II production, both of which contribute to vascular remodeling and heightened arterial resistance [64]. Moreover, dysbiosis-induced alterations in the gut–brain axis lead to overactivation of the sympathetic nervous system (SNS), increasing vasoconstriction and impairing baroreflex sensitivity, further driving hypertensive pathology [65]. Given the strong association between gut dysbiosis, hypertension, and vascular disease, strategies aimed at restoring microbial balance, such as fiber-rich diets, probiotics, and microbiome-targeted therapies, hold promise in mitigating vascular dysfunction and reducing cardiovascular risk. Ongoing research into the gut microbiota’s role in hypertension may pave the way for novel therapeutic interventions targeting the microbiome to improve vascular health.

**Figure 3:**
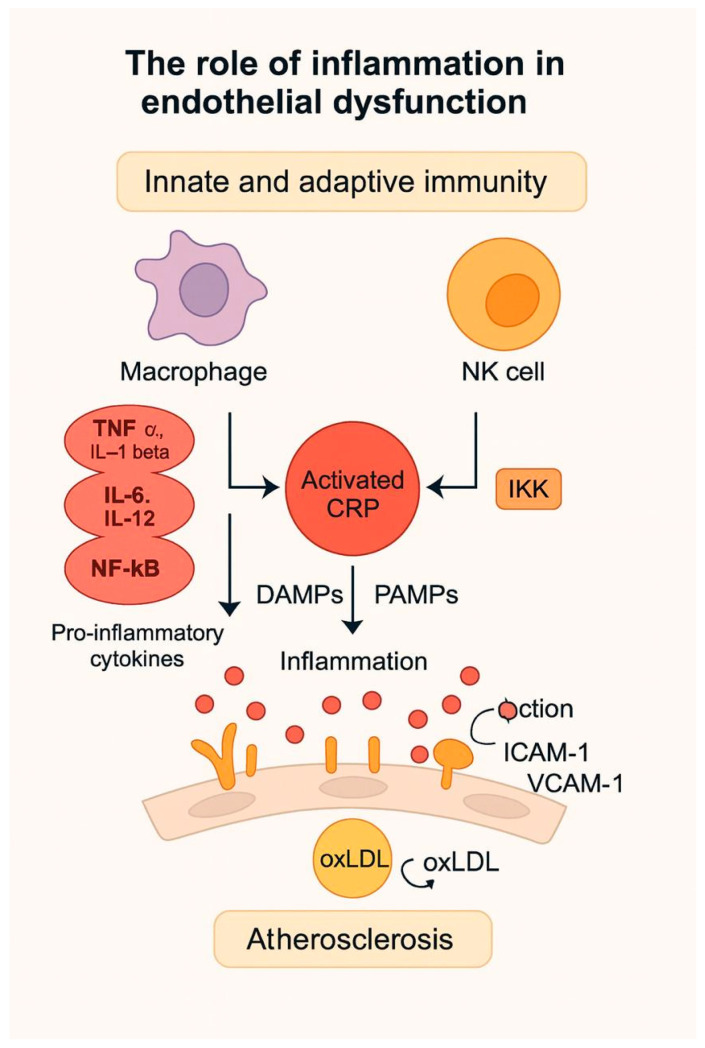
Inflammatory mechanisms in endothelial dysfunction and atherosclerosis.

The Figure 3 illustrates how immune cell activation promotes endothelial inflammation through cytokines such as TNF-α, IL-1β, IL-6, and IL-12, leading to CRP activation and endothelial dysfunction. These processes enhance adhesion molecule expression and contribute to the initiation and progression of atherosclerotic lesions.

### 3.4. Dysbiosis and Ischemic Diseases

Emerging evidence highlights a pivotal contribution of gut microbiota dysbiosis to the onset and progression of ischemic diseases, including coronary artery disease (CAD), myocardial ischemia/reperfusion (I/R) injury, and ischemic stroke. Although these conditions differ in clinical presentation and affected vascular territories, they share common mechanistic pathways, such as chronic inflammation, endothelial dysfunction, oxidative stress, altered immune activation, and disrupted metabolic homeostasis, all of which are substantially modulated by intestinal microbial composition and function.

In patients with CAD, dysbiosis is characterized by a reduction in microbial diversity, a decline in beneficial SCFA-producing bacteria, and an overrepresentation of pro-inflammatory taxa [66]. These alterations correlate with systemic metabolic disturbances, increased oxidative stress, and heightened inflammatory signaling, which collectively contribute to endothelial dysfunction and atherosclerotic progression. Gut-derived metabolites, including TMAO, bile acids, and endotoxins, play a central role in these processes by promoting vascular inflammation, impairing nitric oxide bioavailability, and enhancing thrombogenic activity, mechanisms that consolidate the link between intestinal dysregulation and coronary ischemic events [66,67]. The gut-heart axis has also been implicated in determining the severity of myocardial injury during I/R episodes. Experimental studies demonstrate that dysbiosis disrupts intestinal barrier integrity and promotes systemic translocation of inflammatory mediators, thereby amplifying myocardial oxidative stress, immune activation, and cardiomyocyte apoptosis following reperfusion [68]. Modulation of the gut microbiota in preclinical models can attenuate inflammatory responses and reduce infarct size, underscoring the mechanistic importance of gut-derived signals in myocardial I/R pathophysiology [68].

A similarly robust body of evidence supports the role of dysbiosis in ischemic stroke. Clinical and translational studies show that stroke patients exhibit profound imbalances in microbial composition, including reductions in SCFA-producing species and expansion of pro-inflammatory bacteria [69,70,71]. These alterations may influence cerebrovascular risk by promoting endothelial dysfunction, increasing systemic inflammation, and affecting the stability of atherosclerotic plaques in extracranial and intracranial arteries [71]. In large-artery atherosclerotic stroke and transient ischemic attack, a distinct pattern of dysbiosis has been observed, characterized by altered microbial diversity and significant changes in circulating microbial metabolites, including reduced TMAO levels [72]. Following an ischemic event, the gut–brain axis undergoes bidirectional disruption: stroke-induced autonomic dysregulation and intestinal hypoperfusion contribute to further microbial imbalance, which in turn exacerbates post-stroke systemic inflammation, compromises blood–brain barrier integrity, and influences neurological outcomes [73,74]. These reciprocal interactions highlight a dynamic relationship in which dysbiosis is not only a contributor to stroke development but also a determinant of post-stroke recovery and long-term prognosis [73,74].

Overall, the accumulating data indicate that dysbiosis plays a central role in the pathophysiology of ischemic diseases through interconnected inflammatory, metabolic, and vascular mechanisms. These findings open new avenues for microbiota-targeted therapeutic strategies aimed at modulating ischemic risk, attenuating tissue injury, and improving recovery across the spectrum of ischemic cardiovascular and cerebrovascular conditions.

## 4. Nutraceuticals, Vascular Health and Microbiota

Nutraceuticals is a broader term and includes bioactive supplements that, based on their structure, origin, or properties, can be classified into probiotics, prebiotics, polyphenols (flavonoids and non-flavonoids), omega-3 fatty acids, carotenoids, vitamins, minerals [75,76].

### 4.1. Polyphenols

#### 4.1.1. Resveratrol

Resveratrol (3,5,4′-trihydroxytrans stilbene) is a polyphenol, a hydroxylated compound of stilbene. There are two isomeric forms (cis and trans), but only one (trans-resveratrol) has cardioprotective benefits [77]. This compound is found in high concentrations, especially in grapes, berries, and nuts which is known for its important role in reducing the risk of atherosclerosis [78]. Even though the absorption of resveratrol is high (approximately 70%), bioavailability is low (20%) because most of the absorbed resveratrol is rapidly transformed in epithelial cells, extensively metabolized in the liver, and then eliminated through the kidneys [79].

The endothelium is known to be the primary regulator of vascular tone and vascular pressure and resveratrol has been shown to increase endothelial NO production by upregulating the expression of eNOS, which is a constitutive enzyme in the endothelium, decreasing endothelin-1 synthesis and endothelial oxidative stress [80]. This effect results from sirtuin-1 (SIRT1) activation, either through a substrate-dependent mechanism or indirectly by inhibiting phosphodiesterases or enhancing the effect of lamin A. SIRT1 stimulates eNOS activity [81]. Some of the studies that have demonstrated the beneficial effect of resveratrol in vascular diseases are presented in Table 1.

#### 4.1.2. Quercetin

Quercetin belongs to flavonoid compounds, it is a natural polyphenol found in apples, onions, tea leaves, mango, and has hydrophobic properties that limit its absorption. Thus, various dosage forms or delivery systems have been developed to improve bioavailability [82,83]. Regarding cardiovascular diseases, quercetin plays an important role in the prevention and treatment of atherosclerosis. This flavonoid has the ability to block nicotinamide adenine dinucleotide phosphate (NADPH) oxidase, activate protein kinase (AMPK) pathway, stimulate endothelial nitric oxide synthase (eNOS), reduce matrix metalloproteinases 1 (MMP-1), and decrease the serum lipids (cholesterol and triglycerides levels). These functions lead to antioxidant, vasodilator, and anti-inflammatory properties, preventing the formation of atherosclerotic plaque or, if it does form, quercetin can stabilize it [84,85]. There is a bidirectional relationship between quercetin and the gut microbiota. On the one hand, some strains of intestinal bacteria (Bacteroides fragilis, Clostridium perfringens, Eubacterium ramulus) can transform quercetin into metabolites with anti-inflammatory, antiatherosclerotic or antidiabetic effects. On the other hand, quercetin can influence the intestinal composition, increasing the abundance of Bifidobacterium, Bacteroides, Clostridia, or Lactobacillus and suppressing the growth of Enterococcus and Fusobacterium [86]. The role of quercetin in vascular diseases has been highlighted in various studies (Table 1).

#### 4.1.3. Curcumin

Curcumin is a natural polyphenol extracted from turmeric (*Curcuma longa* L.), a dietary supplement used as a remedy in traditional medicine in China and India for centuries [87]. The chemical structure of curcumin gives it the properties to interact with various cytokines and enzymes such as cyclooxygenase-2 (COX 2), inducible nitric oxide synthase (iNOS), lipoxygenase, and malondialdehyde (MDA), leading to antioxidant, anti-inflammatory, or antiangiogenic effects [88]. The use of curcumin has been limited by its poor water solubility and bioavailability [89]. After administration, it is biotransformed in the liver and intestinal cells and also in the gut under the action of intestinal microbiota. Some bacteria in the gut, through hydroxylation, demethylation, and reduction, can transform curcumin into active metabolites with anti-inflammatory, antioxidant, cardio or neuroprotective effects [90]. On the other hand, curcumin has the ability to influence the diversity and abundance of some bacteria in the gut: it decreases the abundance of Blautia spp., and Bacteroides, increases the abundance of Lactobacillus and reduces the ratio of Firmicutes/Bacteroidota [91,92]. Zhang et al. demonstrated that curcumin decreased plasma levels of IL-1β, TNF-α, soluble VCAM-1, ICAM-1, and inhibited TLR4 expression [93]. Curcumin can inhibit NF-κB and Nrf2 pathways, activate JAK2/STAT3, PI3K-Akt and ERK1/2 pathways, and downregulate IL-8 expression [94,95,96] (Table 1).

### 4.2. Omega-3 Polyunsaturated Fatty Acids

Omega-3 polyunsaturated fatty acids (PUFAs) are essential fats that primarily provide heart benefits. They are found in marine fish species, some algae, and also in plant-based sources such as flaxseeds, chia seeds, and walnuts [97]. These acids interfere with the gut microbiota by increasing the abundance of beneficial bacteria, leading to a high amount of SCFAs, which are known for their role in reducing inflammation, modulating metabolic activity, and immune function [98]. The vascular protective effects of omega 3 PUFA involving the microbiota are achieved through: inhibition of NF-κB, iκB, reduction of proinflammatory cytokines TNF a, IL-6, IL-1B, decrease in TMA, increase in butyrate—an important SCFA with anti-inflammatory properties [99]. The influence of omega-3 PUFAs on the microbiota seems to be related to the source: flaxseeds decrease the abundance of Bacteroidetes and those from fish decrease Firmicutes. Thus, they have the ability to reduce LPS-producing bacteria and reduce endotoxemia [100] (Table 1).

### 4.3. Probiotics

Probiotics are known for several benefits, including those at the vascular level by decreasing inflammation, improving lipid profiles, regulating blood pressure, and improving endothelial function. Probiotics are found in numerous fermented products that include bacteria (e.g., *Lactobacillus*, *Bifidobacterium*) or yeasts (e.g., *Saccharomyces*). The beneficial effects of probiotics are primarily related to the inhibition of pathogenic bacteria (*Enterococcus faecalis*, *Listeria monocytogenes*, *Staphylococcus aureus*, *Escherichia coli*) and the stimulation of the growth of beneficial bacteria [101].

Probiotics can modulate innate and adaptive immunity by decreasing proinflammatory cytokines (TNF-α, IL-1, IL-6) and can influence the composition of the intestinal microbiota, modifying the Firmicutes/Bacteroidetes ratio and other bacterial species involved in the production of fatty acids, LPS, TRP metabolites, TMAO or SCFA. Thus, probiotics exert a positive impact on cardiovascular health [102,103] (Table 1).

**Table 1:** Effect of nutraceutical agents on microbiota and vascular outcomes.

Nutraceutical Agent	Study Model	Important Finding	Effect on Microbiota and Their Metabolite	References
**Resveratrol**	animal model/mice	- inhibited TMAO synthesis - decreased TMAO-induced ATS in mice	- increased the abundance of *Bacteroides*, *Lactobacillus*, *Bifidobacterium*, and *Akkermansia* - inhibited the growth of *Enterococcus faecalis* - decreased the abundance of *Prevotella*, *Ruminococcaceae* - increased *Bacteroidetes/Firmicutes* ratio	[81]
**Resveratrol Propionate Ester**	Animal model/Sprague Dawley rats	- attenuated hypertension in juvenile CKD rats treated with adenine - increased NO availability - decreased levels of both symmetric and asymmetric dimethylarginine (SDMA and ADMA), the inhibitors of NOS - increased levels of SCFA	- increased the abundance of *Eubacterium*, *Bacteroides*, *Ruminococcus*, *Ligilactobacillus*, and *Allobaculum* - decreased the abundance of *Parabacteroides*, *Peptococcus*, and *Turicibacter*	[104]
**Resveratrol Butyrate Monoester**	Animal model/Sprague Dawley rats	- improved hypertension in the adenine-fed group of rats - attenuated the reduction in plasma L-arginine concentration in the adenine-fed group of rats - prevented the decrease in NO bioavailability - suppressed oxidative stress	- increased abundance of the genera *Duncaniella* and *Ligilactobacillus* and *Monoglobus* - decreased the abundance of *Eubacterium*	[105]
**Resveratrol**	- clinical trial pilot, randomized, placebo-controlled - men with metabolic syndrome	- no change in SIRT gene expression in adipose tissue - improved insulin sensitivity and glucose tolerance during a 2 h oral GTT, in Caucasian subjects	- altered alpha and beta diversity - significantly increased levels of *Akkermansia muciniphila*, *Fusobacteria*, and *Megamonas*, compared to Caucasian subjects	[106]
**Quercetin**	animal model/mice	- decreased the areas of atherosclerotic lesions in the aortic sinus - reduced the plaque sizes and lipid contents - decreased the level of malondialdehyde - increased the level of IL-6	- enhanced alpha diversity - increased the abundance of *Actinobacteria* and *Bacteroidetes* - decreased the abundance of *Firmicutes*	[107]
	animal model/mice	- significantly reduced total cholesterol and triglyceride levels in high-fat diet-induced dyslipidemia - protected blood vessels from changes induced by high-fat diet - prevented plaque formation in the artery	- no differences were observed between groups at the phylum levels (*Bacteroidetes*, *Firmicutes* and *Proteobacteria*)	[108]
**Curcumin**	animal model/mice	- reduced plasma LDL, TG, and T-CHO concentrations - increased plasma HDL concentration - down-regulated the expression of NF-κB p65 and NLRP3 - reduced level of IL-1β and IL-6	- increased *Verrucomicrobia* and *Akkermansia* abundance - decreased *Lactobacillus* abundance	[109]
**Omega-3 PUFAs**	animal model/rats	- prevented the increase in norepinephrine - decreased blood pressure - increased plasma butyrate levels	- decreased in *Firmicutes/Bacteroidetes* ratio - increased the abundance of *Roseburia*, *Ruminococcaceae*	[110]
**Omega-3 PUFAs**	animal model/rabbits	- increase of plasma TMAO - reduced levels of hepatic FMO3 - significantly reduced levels of inflammatory genes *MCP-1*, *TGF-β*, and *IL-1*	- decreased *Clostridia* abundance - increased *Bacteroides*, *Alistipe*, *Roseburia*	[111]
**Probiotics** ***B. lactis M8 and L. rhamnosus M9***	animal model/mice	- reduced BP levels - regulated the production of SCFAs	- increased levels of *Pyrolobus*, *Coprobacillus* and *Butyricimonas*, *Allobaculum* - reduced levels of *Abiotro.phia*, *Anaerostipes*, *Alloprevotella*	[112]
**Probiotics** ***Romboutsia lituseburensis JCM1404***	animal model/rats	- ameliorated endothelial function - increased the metabolism of adipocytes - suppressed adipogenesis	- decreased levels of *Romboutsia and Clostridium_sensu_stricto_1*	[113]
**Probiotics** ***Limosilactobacillus reuteri***	animal model/ mice (preeclampsia mice model induced by NO blockade)	- alleviated hypertension, gut dysbiosis, and endothelial dysbiosis	- reduced abundance of *Proteobacteria*	[114]

**Abbreviations:** TMAO—trimethylamine N-oxide; ATS—atherosclerosis; CKD—chronic kidney disease; NO—nitric oxide; SCFA—short-chain fatty acids; SIRT—sirtuin; GTT—glucose tolerance test; IL-6—interleukin 6; LDL—low-density lipoprotein; TG—triglyceride; T-CHO—total cholesterol; HDL—high-density lipoprotein; NF-κB p65—nuclear factor NF-kappa-B p65; NLRP3—nucleotide-binding domain, leucine-rich-containing family, pyrin domain-containing-3; IL-1β—interleukin 1β; PUFAs—polyunsaturated fatty acids; FMO3—flavin-containing monooxygenase 3; MCP-1—monocyte chemoattractant protein-1; TGF-β—transforming growth factor beta; IL-1—interleukin 1; BP—blood pressure.

### 4.4. Plant-Derived Essential Oils and Their Constituent Phytochemicals (Table 2)

Essential oils (EOs) stand out as natural modulators of the gut microbiota due to their selective antimicrobial and antioxidant properties. Many EOs exhibit higher inhibitory concentrations for probiotic strains than for pathogenic bacteria, allowing them to suppress harmful microorganisms while maintaining or enhancing the viability of beneficial species, such as Lactobacillus and Bifidobacterium. This selectivity makes the combined use of EOs and probiotics a promising strategy for managing intestinal infections and supporting microbial balance [115,116]. When incorporated into fermented foods such as yogurt, probiotic beverages fortified with EO may improve probiotic survival during storage and gastrointestinal transit. Studies show that EOs (e.g., mint, basil, eucalyptus, etc.) enhance probiotic viability while increasing antioxidant activity—conditions that promote a healthier gut microbial environment. Probiotic beverages fortified with EO also demonstrate potent inhibitory effects against common enteric pathogens, suggesting their potential to prevent dysbiosis [115,117]. Also, the complex phytochemical composition of the essential oil—rich in terpenes, phenols, and flavonoids—confers antioxidant, anti-inflammatory, vasodilator, and endothelial-protective activities that can positively influence vascular function [118,119].

**Table 2:** Vascular effects of plant-derived essential oils.

Plant Species/Family	Essential Oils Major Compounds	Effect	References
***Elsholtzia ciliata*/Lamiaceae**	○ elsholtzia ketone ○ dehydroelsholtzia ketone	Inhibition of phenylephrine-induced thoracic aortic contraction in rats	[120]
***Rubus ulmifolius*/** **Rosaceae**	○ Tanins—Galloyl-bis-HHDP glucose derivative ○ Flavonoids—afzelin, quercetin-3-O-β-d-glucuronide and kaempferol derivatives	Upregulated - *Amotl2* (Angiomotin-like 2), a gene involved in endothelial cell functions *Tfcp2l1* a gene with potential vasorelaxant effect Determined highly significant vasodilatory effect	[121]
***Foeniculum vulgare*/** **Umbellifers**	○ anethole ○ 1-(4-methoxyphenyl)-2-propanone ○ ethoxydimethylphenylsilane ○ para-anisaldehyde diethyl acetal	Suppressed hypoxia/reoxygenation (H/R) -induced ROS generation, DNA damage and influenced mitochondrial membrane potential in H/R injury model of H9C2 heart myoblast cells	[122]
***Mentha longifolia*/** **Lamiaceae**	○ n—butanolic fraction (thymol, carvacrol, and menthol)	Produced relaxation in the procine coronary artery—direct action on vascular smooth muscle cells (VSMCs) Stimulated of cAMP and cGMP pathways and inhibitied of vascular isoforms of PDEs	[123]
***Citrus aurantifolia*/** **Rutaceae**	○ D-limonene ○ γ-terpinene ○ terpinolene ○ α-terpineol	○ inhibited VSMC proliferation ○ inhibited the MAPK/ERK and PI3K/AKT/mTOR signaling pathways	[124]
***Zingiber officinale*/Zingiberaceae**	○ citral	○ ameliorated atherosclerosis in ApoE^−/−^ mice ○ suppresed TMA (trimethylamine) and TMAO (trimethylamine-N-oxide) and remodelated gut microbiota composition ○ lowered plasma IL-1β, TNF-α, glucose, and insulin levels	[125]
***Origanum*, *Satureja*, *Thymbra*, *Thymus*, and *Corydothymus*/** **Labiatae**	○ carvacrol	✓ in the myocardical infarction model: ○ reduced levels of troponin T, BNP, IL-6, ○ decreased diastolic blood pressure and heart rate ○ reduced necrosis, oedema and mononuclear cell infiltration in cardiac tissue	[126]
***Coptis*/** **Ranunculaceae**	○ berberine	○ decreased the levels of TMA and TMAO in faeces and blood ○ improved blood lipid profiles ○ interrupted atherosclerotic plaque formation in blood vessels in the HFD-fed hamsters	[127]
***Allium sativum/*** ***Amaryllidaceae***	○ organosulfur compounds (diallyl disulfide, methyl allyl disulfide, methyl allyl trisulfide, diallyl trisulfide, and diallyl tetrasulfide)	○ reduced total cholesterol and LDL-C levels ○ elevated HDL-C level ○ downregulated NF-κB and TNF-α expression ○ improved nitric oxide availability ○ significant cardioprotective effects	[128]
***Olea europaea*/** **Oleaceae**	○ oleic acid ○ oleacein	○ dilated resistance mesenteric artery in a dose-dependent manner ○ activated L-type Ca^2+^ channels and GPCR-Gαi-mediated-PLC pathways	[129]

**Abbreviations:** ApoE^−/−^—apolipoprotein E knockout mice; Amotl2—Angiomotin-like 2; BNP—B-type natriuretic peptide; cAMP—cyclic adenosine monophosphate; cGMP—cyclic guanosine monophosphate; GPCR—G-protein-coupled receptor; H9C2—rat cardiomyoblast cell line H9C2; HFD—high-fat diet; H/R—hypoxia/reoxygenation; HDL-C—high-density lipoprotein cholesterol; IL-1β—interleukin-1 beta; IL-6—interleukin-6; LDL-C—low-density lipoprotein cholesterol; MAPK/ERK—mitogen-activated protein kinase/extracellular signal-regulated kinase; mTOR—mammalian target of rapamycin; NF-κB—nuclear factor kappa-light-chain-enhancer of activated B cells; PDEs—phosphodiesterases; PI3K/AKT—phosphoinositide-3-kinase/protein kinase B; PLC—phospholipase C; ROS—reactive oxygen species; TMA—trimethylamine; TMAO—trimethylamine N-oxide; TNF-α—tumor necrosis factor alpha; VSMC/VSMCs—vascular smooth muscle cell.

## 5. Nutraceuticals and Their Impact on Vascular Health

Nutraceuticals can have a positive impact on vascular health by influencing the main mechanisms that are involved in vascular diseases: oxidative stress, inflammation, and endothelial dysfunction.

### 5.1. Oxidative Stress

One of the major factors leading to the development of atherosclerosis and hypertension is oxidative stress. Factors such as sestrins or uncoupling protein 2 (UPC2) influence the effect of oxidative stress on the vascular system [130]. Sestrins are proteins that respond to various environmental challenges, including oxidative ones. They influence several signaling pathways: upregulate AMPK, regulate mTOR (mammalian target of rapamycin) complexes, insulin-AKT complexes, and redox signaling pathways. The first studies of sestrins as antioxidants were made due to their sequence similarity to that of a bacterial oxidoreductase AhpD. This sequence can reduce alkyl hydroperoxide radicals, thus directly regulating ROS. Furthermore, these proteins are direct targets of Nrf2, an antioxidant transcription factor, and in this way enhance the antioxidant capacity of cells [131,132,133].

UPC2 is a ubiquitously expressed protein with a known role in preventing oxidative stress [134]. Regarding vascular dysfunctions, UPC2 is considered an important protective factor by reducing mitochondria-derived ROS, mitochondria being a key contributor to the pathogenesis of vascular diseases [135,136]. This protein, which acts as an anion carrier, in addition to generating ROS, can influence processes such as mitochondrial membrane potential or calcium homeostasis. Experimental studies have shown that UCP2 expression can be activated by AMPK/PPARalpha and thus exerts protective effects in experimental vascular models (e.g., ischemic stroke) [137].

### 5.2. Inflammatory Effects

A key role in vascular inflammation is played by the interaction between immune and vascular cells [138]. Endothelial inflammation is correlated with the activity of innate and adaptive immunity. Macrophages contribute to the release of pro-inflammatory cytokines such as TNF alpha, Il-1 beta, IL-6, and IL-12. These cytokines activate NF-κB which stimulates endothelial adhesion molecules E-selectin, P-Selectin, VCAM-1, ICAM-1 expression, and chemokines like MCP-1 (monocyte chemoattractant protein). VCAM-1 expression in the plasma membrane is influenced by transcription induced through the IKK/NF-κB pathway [139,140]. In several studies, levels of VCAM-1, ICAM-1, and E-selectin have been correlated with inflammatory processes in the endothelium (e.g., overexpression of VCAM-1 and ICAM-1 in the early stages of atherosclerosis) [141,142].

C-reactive protein (CRP) has the ability to interact with DAMPs (damage-associated molecular patterns), molecules released during cellular stress or tissue injury, and PAMPs (pathogen-associated molecular patterns) molecules derived from microorganisms (e.g., lipopolysaccharides), resulting in the activated form of CRP that activates the complement cascade and the M1 inflammatory macrotype. As a result, these stimuli facilitate endothelial deposition of oxLDL, activation of inflammasomes, maintenance of inflammation, release of endothelial adhesion molecules, and ultimately disruption of vascular endothelial homeostasis. Also, CRP may downregulate eNOS in endothelial cells and decrease NO [143,144].

To date, three isoforms of CRP have been described: CRP (pentamer), mCRP (monomer), and transitional CRP. The effects on inflammation of the CRP isoforms appear to be different; pCRP has anti-inflammatory effects, while mCRP has pro-inflammatory effects. The latter isoform stimulates platelet aggregation, neutrophil migration, activates the release of pro-inflammatory cytokines, and stimulates NK cell activity [145,146,147]. Recently, Melnikov and colab., in a clinical study demonstrated that elevated levels of mCRP were associated with coronary heart disease [146].

### 5.3. Endothelial Dysfunction

Endothelial dysfunction is strongly linked to factors that influence vascular tone, inflammation, and oxidative stress. The imbalance between endothelium-derived relaxing factors and endothelium-derived contracting factors in favor of the latter predisposes endothelial dysfunction. NO and prostacyclin have a protective effect, while endothelin -1 and superoxide anion have a detrimental effect on vascular tone [148].

The main cause of endothelial dysfunction is related to the reduction of NO bioavailability. This signaling molecule is produced by three isoforms of NO synthase: nNOS (neuronal), iNOS (inducible) and eNOS (endothelial) [149,150]. NO synthesis starts from L-arginine in endothelial cells and uses cofactors such as NADPH, oxygen, or tetrahydrobiopterin. After synthesis, NO reaches vascular smooth muscle cells, converts GTP to cGMP by activating guanylate cyclase, and then relaxes the cells at this level by removing calcium [142]. Thus, factors that influence the NO synthesis reaction (reduction in L-arginine availability, eNOS phosphorylation, increased NO scavenging, reduced eNOS expression, decreased cofactors), may influence the bioavailability of this small molecule. Several nutraceuticals and their impact on vascular health are presented in Table 3 [151].

In addition to the main nutraceutical categories discussed—such as polyphenols, omega-3 fatty acids, probiotics, and prebiotics—vitamins and minerals also contribute to vascular protection, partly through antioxidant, anti-inflammatory, and endothelial-supporting mechanisms [162].

Vitamins represent a fundamental category of nutraceuticals with the capacity to influence both gut microbiota composition and key mechanisms involved in vascular homeostasis [163]. Beyond their classical metabolic roles, many vitamins exhibit anti-inflammatory, antioxidant, and endothelial-protective properties, making them important modulators in the microbiota–vascular health axis [164,165,166].

Evidence indicates that vitamin D and magnesium can enhance flow-mediated dilation and modulate gut microbial composition [167,168], while vitamin K2 is associated with reduced arterial stiffness and may interact with menaquinone-producing bacteria [169].

Vitamins C and E exert important antioxidant actions that support endothelial integrity [170], whereas trace elements such as zinc and selenium influence gut microbial diversity and barrier function, ultimately affecting vascular inflammation [171,172].

## 6. Conclusions

The link between nutraceuticals, microbiota, and vascular diseases is an emerging area of research that explores how the diet, specifically through nutraceuticals, affects the gut microbiota, and how this interaction, in turn, influences the development and progression of vascular diseases. By modulating the gut microbiota through diet, nutraceuticals may help prevent or alleviate vascular diseases, especially by reducing inflammation, oxidative stress, and improving endothelial function. Future research is likely to further elucidate the precise mechanisms behind these interactions and their potential therapeutic implications.

## Figures and Tables

**Table 3:** Nutraceuticals and their impact on vascular health.

Nutraceutical Agents/Sources	Study Model	Important Finding	Conclusion	References
**Epicatechin/green tea and cocoa**	Animal model/rat model of metabolic syndrome	- significantly increased serum NO - significantly decreased triglycerides, LDL cholesterol - significantly reduced serum levels of IL-6 and 8-isoprostane All findings were in epicatechin-treated HFHC rats vs. control	- protect against electrical dysfunction and enhance vascular reactivity in rat models of metabolic syndrome - did not affect blood pressure	[152]
	Animal model/deoxycorticosterone acetate (DOCA)-salt hypertensive rats	- significantly reduced MDA	- significantly reduced blood pressure - prevented cardiac compliance and myocardial stiffening	[153]
	Clinical trial/double-blind, placebo-controlled/healthy male	- had no significant effects on nitrites or nitrates (considered by some authors not the best markers for NO bioavailability).	- improved vascular function in a dose-dependent manner	[154]
***Arthrospira platensis*** **(Spirulina)**	Animal model/pig model of ST-elevation myocardial infarction	- increased iNOS in the infarcted myocardium - decreased myocardial MCP-1 expression - attenuated DNA-oxidative damage	- reduced infarct size - improved cardiac function	[155]
	Animal model/age-induced vascular dysfunction in rats	- increased the release of NO - decreased the production of superoxide - increased expression of p-eNOS and HO-1	- improved the vasomotor response in the aorta of aged rats - increased ACh-induced relaxation	[156]
	Animal model/rat model of hypercholesterolemia	- increased in the levels of the primary bile acid cholic acid in the faeces - decreased in the levels of deoxycholic acid in the faeces - reduced the increase in total cholesterol and LDL-cholesterol induced by high fed diet	- anti-hyperlipidaemic effect	[157]
***Allium cepa***	Animal model/rats fed with high fed-diet	- decreased total cholesterol, LDL-cholesterol, triglycerides, creatinine kinase - increased HDL-cholesterol	- decreased Castelli risk index (or cardiac risk ratio that reflects the formation of coronary plaque)	[158]
***Nigella sativa***	Nonrandomized clinical trial	- decreased total cholesterol, LDL-cholesterol, triglycerides, and LDL-C/HDL-C ratios - increased HDL-cholesterol	- reduced systolic blood pressure, diastolic blood pressure, mean arterial pressure, heart rate	[159]
**Zinc**	Animal model/zinc deficiency-induced hypertension	- modulation of renal Na^+^ transport - renal Na^+^Cl^−^ cotransporter is a Zn 2^+^ regulated transporter that is upregulated by zinc deficiency	- regulated blood pressure induced by zinc deficiency	[160]
**C Vitamin**	Clinical trial/patients with hypertension	- decreased production/activity of oxygen-derived free radicals that contribute to endothelial dysfunction	- improved impaired endothelium-dependent vasodilatory function (in human coronary arteries)	[161]

**Abbreviation:** NO—nitric oxide; LDL—low-density lipoprotein; HDL—high-density lipoprotein; IL-6—interleukin 6; HFHC—high-fat-high-carbohydrate; MDA—malondialdehyde; iNOS—inducible nitric oxide synthase; MCP-1—monocyte chemoattractant protein-1; p-eNOS—phosphorylated endothelial nitric oxide synthase; HO-1—heme oxygenase 1.

## Data Availability

No new data were created or analyzed in this study. Data sharing is not applicable to this article.

## Abbreviations

The following abbreviations are used in this manuscript:

AMPK	AMP-activated protein kinase
APC	Antigen-presenting cell
Baf60a	Transcriptional coactivator Baf60a
cGMP	Cyclic guanosine monophosphate
COX-2	Cyclooxygenase 2
CRP	C-reactive protein
CVD	Cardiovascular diseases
DAMPs	Damage-associated molecular patterns
eNOS	Endothelial nitric oxide synthase
ERK1/2	Extracellular signal-regulated kinases 1 and 2
FMT	Fecal microbiota transplantation
GALT	Gut-associated lymphoid tissue
GBD	Global Burden of Disease
GTP	Guanosine triphosphate
ICAM-1	Intercellular adhesion molecule 1
IL-1β	Interleukin 1 beta
IL-6	Interleukin 6
IL-8	Interleukin 8
iNOS	Inducible nitric oxide synthase
JAK2	Janus kinase 2
LPS	Lipopolysaccharide
MDA	Malondialdehyde
MMP-1	Matrix metalloproteinase 1
mCRP	Monomeric C-reactive protein
mTOR	Mechanistic target of rapamycin
NF-κB	Nuclear factor kappa-light-chain-enhancer of activated B cells
Nrf2	Nuclear factor erythroid 2–related factor 2
nNOS	Neuronal nitric oxide synthase
NO	Nitric oxide
pCRP	Pentameric C-reactive protein
PAMPs	Pathogen-associated molecular patterns
PI3K	Phosphoinositide 3-kinase
PUFAs	Polyunsaturated fatty acids
RAS	Renin-angiotensin system
ROS	Reactive oxygen species
SCFAs	Short-chain fatty acids
SIRT1	Sirtuin 1
SNS	Sympathetic nervous system
STAT3	Signal transducer and activator of transcription 3
TMA	Trimethylamine
TMAO	Trimethylamine N-oxide
TLR4	Toll-like receptor 4
TNF-α	Tumor necrosis factor alpha
TRP	Tryptophan (metabolites)
UCP2/UPC2	Uncoupling protein 2
VCAM-1	Vascular cell adhesion molecule 1
